# The Vomeronasal Organ: A Neglected Organ

**DOI:** 10.3389/fnana.2017.00070

**Published:** 2017-08-21

**Authors:** Biagio D'Aniello, Gün R. Semin, Anna Scandurra, Claudia Pinelli

**Affiliations:** ^1^Department of Biology, University of Naples “Federico II,” Naples, Italy; ^2^William James Center for Research, Instituto Superior de Psicologia Aplicada - Instituto Universitário (ISPA-IU) Lisbon, Portugal; ^3^Department of Environmental, Biological and Pharmaceutical Sciences and Technologies, University of Campania “L. Vanvitelli,” Caserta, Italy

**Keywords:** olfaction, human VNO, VNO homolog, vertebrate VNO, vomeronasal organ

Even though the vomeronasal organ, part of the accessory olfactory system (AOS), has been extensively studied in vertebrates, a lot remains to be understood on its function (see Spehr et al., 2006). The problematic nature of our understanding of this structure does not only relate to its function, but also partly to its presence/absence in vertebrates. A recent article in “Science” (McGann, 2017) has denied the presence of the VNO in humans. It is ironic that the first indication of a VNO was in humans and only at a later stage in other mammals. Ruysch, a seventeenth to eighteenth century Dutch anatomist, was the first to mention an organ near the nasal septum of a human infant (Ruysch, 1703, 1724). However, the discovery of the human VNO was ascribed to Kölliker (1877) as Ruysch did not supply an accurate description or provide a name and according to Bhatnagar and Smith (2003) he described a “nasal canal,” too close to the palate to represent the VNO. Thus, it was Kölliker (1877), who among eighteenth to nineteenth century investigators became known as the first to provide evidence of the human VNO as a histologically identifiable structure. Indeed, he identified this structure in both the fetus and the adult human brain. Jacobson (1811) studied the VNO intensively across a variety of mammals, although he denied its existence in humans. Consequently, VNO is now also known as “Jacobson's organ,” largely due to Potiquet (1891), who supplied the first extensive discussion of the VNO in humans.

In primates, the VNOs have extremely variable features (Smith et al., 2001). In human embryos, the VNO develops very early (Smith et al., 1997; Garrosa et al., 1998). The nerve fibers of the VNO extend together with a cluster of migrating gonadotropin releasing hormone (GnRH)-secreting cells from the olfactory placode toward the brain (Wray, 2010). Subsequently, compared to other mammals the VNO of the adult human shows some signs of regression (Trotier et al., 2000; Bhatnagar and Smith, 2001; Trotier, 2011).

Endoscopic observations of humans show that it is possible to observe the vomeronasal cavities and ducts in the case of *some* individuals (Moran et al., 1991; Stensaas et al., 1991; Boehm and Gasser, 1993; Trotier and Døving, 1996; Trotier et al., 2000; Witt and Hummel, 2006; Stoyanov et al., 2016). In adult humans, the VNO is structurally a tube-shaped canal with a blind-ending opening into the nasal cavity (Bhatnagar and Smith, 2001). According to Bhatnagar and Smith (2001), the presence and the location of the VNO is clearly demonstrated by serial sectioning of the nasal septum convincingly. However, it lacks sensory neurons and nerve fibers (Trotier et al., 2000; Trotier, 2011; Stoyanov et al., 2016). The size and shape of the VNO exhibits considerable variability in humans.

The functional aspects of the human vomeronasal organ are the subject of debate. In humans, the genes coding for vomeronasal receptor proteins and the specific ionic channels involved in the transduction process identified in species with a functional VNO have mutated and are non-functional. Furthermore, in the case of humans, no accessory olfactory bulbs (AOB) that receive information from the vomeronasal receptor cells are present. Thus, the sensory function of the vomeronasal is considered to be non-operative (see Dulac and Torello, 2003). Notably, it has recently been shown that there are morphological connections of the VNO cells with the underlying capillaries. These, along with the expression of calcium-binding protein in part of these cells, suggest a potential endocrine activity (Wessels et al., 2014). If so, we would have the first evidence of an alternative function than the usually assumed pheromone sensing one for the VNO. An endocrine function of the VNO has not been reported for any other organism and could possibly account for the enigmatic effects of pheromones on human behavior ensuing upon the stimulation of the adult human VNO despite the absence of olfactory sensory neurons (Monti-Bloch et al., 1998).

In most land vertebrates, the associated central nervous structures of the VNO consist of the AOB and the vomeronasal amygdala. The connections between them are established by the corresponding nerves and tracts (Salazar et al., 2016). The glands associated with the VNO secrete fluids that fill this discrete structure (Meredith, 1994; Rehorek et al., 2000; Nowack and Wöhrmann -Repenning, 2009). The specific openings under the palatine vault and the ducts that break into the nasal canal permit the entrance of chemosignals to the VNO. Unlike the cilia of the olfactory receptors to the main olfactory system (MOS) the receptor neurons of the VNO sensory epithelium possess apical microvilli (Stensaas et al., 1991; Døving and Trotier, 1998). Notably, there are also documented cases of ciliated vomeronasal receptor cells (Adams and Weikamp, 1984; Saint Girons and Zylberberg, 1992). The axons of the VNO merge together, forming vomeronasal nerves which end in the AOBs (Barber and Raisman, 1974), within vomeronasal glomeruli.

Even though there are many studies addressing the functional, ontogenetic and phylogenetic aspects of VNO, the literature reveals little agreement. Jacobson's study (1811) underlined that the VNO appeared when the tetrapods conquered the lands. It was logical to link VNO function with the perception of airborne odors (Bertmar, 1981). However, Broman (1920) observed that liquids filled the VNO, which hold for water born substances. For this reason, he hypothesized that the fish main olfactory mucosa was homolog to the VNO of vertebrates, whereas the MOS was a new acquisition of tetrapods. This observation was recently supported by a review in cartilaginous fishes (Chondrichthyes), claiming that the sense of smell could primarily (or completely) rely on the vomeronasal sense (Ferrando and Gallus, 2013). However, this hypothesis seems flawed since the projections of the olfactory bulbs in fish and tetrapods are similar. On one hand, the Bertmar hypothesis is unsustainable given the presence of vomeronasal related structures in larval and the adult aquatic amphibians (Eisthen, 1997). On the other hand, there is increasing evidence reporting the presence of vomeronasal related structures, together with the MOS, in early gnathostomes. In the rat fish (Chimaera, Chondrichthyes), AOBs are described as associated with the dorsal and ventral parts of each main olfactory bulb (Faucette, 1969). In some lungfishes (Dipnoa) species, an AOB has been reported (Rudebeck, 1944; Schnitzlein and Crosby, 1967), with separate vomeronasal and olfactory nerves (Schnitzlein and Crosby, 1967; González et al., 2010). In the ray-finned fishes (Actinopterygii), such as sturgeon (*Scaphirhynchus platorhynchus*; Adair, 1964) and the paddlefish (*Polyodon spathula*; Story, 1964) the AOB has been identified as a dorsomedial part of the main olfactory bulb. Finally, a part of the main olfactory bulbs of *Amia calva*, a living fossil, has been recognized as an AOB (Schnitzlein, 1964). Neither in the chondrichthyans, nor in the osteichthyans a clear VNO has been identified. However, recently a potential vomeronasal organ homolog in form of accessory epithelial crypts within the nasal cavities of dipnoans showing multisynaptic connectivity reminiscent of the tetrapod AOS has been reported (González et al., 2010; Nakamuta et al., 2012; Wittmer and Nowack, 2017). Also, special sensory cells (some microvillous and all crypt cells) with connections supportive of an accessory olfactory or vomeronasal system in teleosts (Biechl et al., 2017) have been described. Thus, it is possible that the VNO may be present in teleosts in an unsegregated form, with the sensory neurons of the VNO mixed with those of the MOS. This hypothesis is supported by the observation that the fish's olfactory mucosa contains both ciliated olfactory sensory neurons (which are typical for the tetrapod main olfactory epithelium) and microvillous olfactory sensory neurons (which are characteristic of VNO in tetrapods) (Hansen et al., 2003, 2004), as well as crypt and Kappe cells which may also contribute to the AOS (Biechl et al., 2017).

Different from fish species, the presence of a VNO has been largely recognized in tetrapods, although, in the proteids (Amphibia, Apoda), in birds and in some mammals (for example cetacea) it is absent. From this overview, it appears that the VNO or vomeronasal equivalents in vertebrates are more widespread than previously believed and the cases of absence in tetrapods are probably secondary adaptive conditions.

## Author contributions

All authors listed have made a substantial, direct and intellectual contribution to the work, and approved it for publication.

### Conflict of interest statement

The authors declare that the research was conducted in the absence of any commercial or financial relationships that could be construed as a potential conflict of interest.
